# An atypical presentation of a bilateral nasolabial cyst: a case report

**DOI:** 10.1093/jscr/rjab017

**Published:** 2021-03-08

**Authors:** Walid Bijou, Rabii Laababsi, Mohamed Amine Mennouni, Youssef Oukessou, Sami Rouadi, Reda Abada, Mohamed Roubal, Mohamed Mahtar

**Affiliations:** ENT Department, Face and Neck Surgery, Hospital August, 20’1953, University Hospital Center IBN ROCHD, Hassan II University, 82, 6, rue lahssen ELAARJOUN quartier des hopitaux, Casablanca, Morocco

## Abstract

The nasolabial cyst is a rare, non-odontogenic, soft tissue cyst that develops submucosally in the anterior nasal floor. This cyst accounts for 0.7% of all non-odontogenic cysts. Bilateral nasolabial cyst represents only 10% of the cases. This cyst originates from the remnants of embryonic nasolacrimal duct tissue. Generally, patients present with swelling and facial deformity and rarely local pain. The definite diagnosis should be based on clinical, radiological and above all histopathologic findings. The treatment is enucleation of the cystic tissue. Following is a case report of a bilateral nasolabial cyst in a 40-year-old woman who presented with a chronic nasal obstruction.

## INTRODUCTION

The nasolabial cyst is also known nasoalveolar cyst, fissural cyst, nasal vestibular cyst and nasal wing cyst. It was first described by Zuckerkandl in 1882 [1]. Currently, it is considered as a developmental, epithelial and non-odontogenic cyst that occurs in the nasal alar region [2]. Its prevalence is estimated to occur in 1.6 per 100 000 persons per year. Most cases occur between the fourth and fifth decades of life. The male to female ratio is 1:3.6. [1]. Patients typically complain of deformity and nasal obstruction. Signs and symptoms include swelling and facial deformity and rarely local pain. The diagnosis is based on clinical, radiological and histopathologic findings. In this paper, due to rarity and atypical presentation, a case of bilaterally located nasolabial cyst is presented.

## CASE PRESENTATION

A 40–year-old woman presented to our department with a chief complaint of bilateral nasal obstruction and rhinorrhea. Neither epistaxis nor pain was noted. The patient had stated her symptoms had been present for over a year. Past medical history was notable for breast cancer treated with surgery and radio chemotherapy under remission.

Extra-oral inspection revealed facial asymmetry, with elevation of the right alar base. There was mild tenderness on palpation.

Anterior rhinoscopy identified a bilateral submucosal smooth fluctuant mass obstructing the nostrils. The right-sided mass measured 2 × 2 cm and the left-sided mass measured 1.5 × 1 cm.

Intra-oral examination revealed bulging of the upper gingivolabial sulcus consistent with the extra-oral findings.

**Figure 1:**
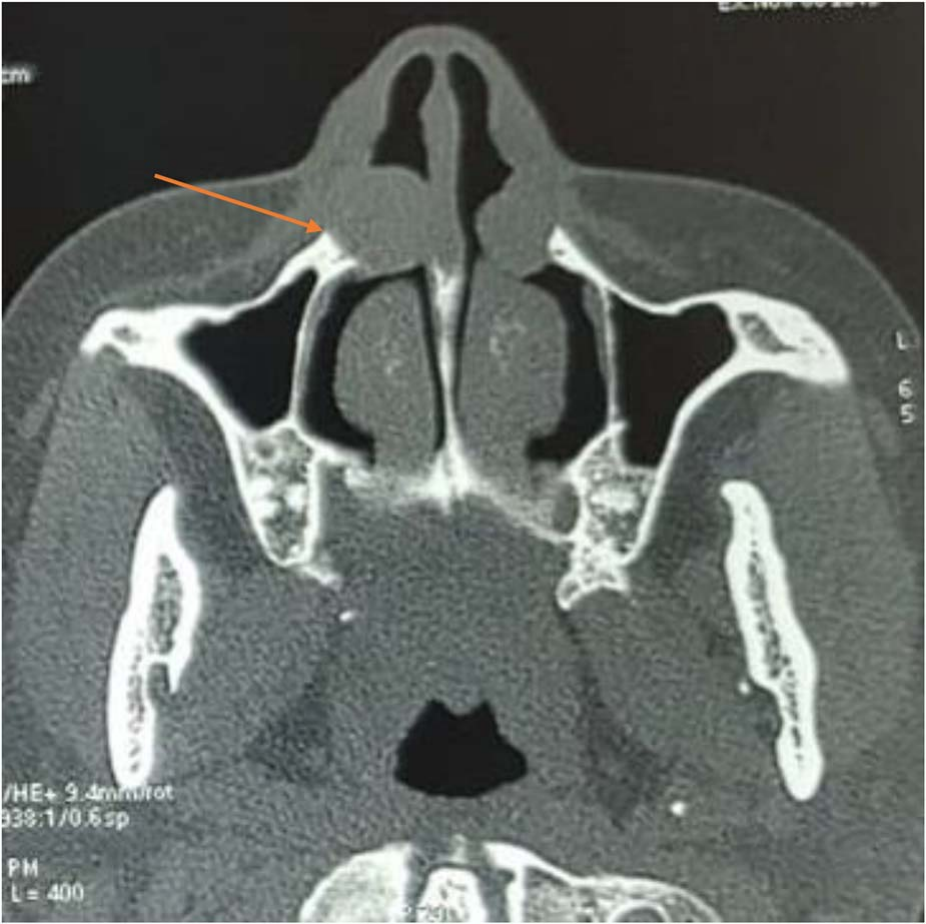
Axial computed tomography showing a bilateral well-defined, hypodense, extraosseous lesion near the ala nasi; note the cortical scalloping on the right side (arrow).

Computed tomography without injection showed bilateral mass on the inferior nasal alar region, measuring 2.1 × 2.2 × 1.9 cm on the right side and 1.7 × 1.5 × 1.2 cm on the left side. The masses were rounded, well defined and exhibiting soft tissue density. There was mass effect upon the maxilla causing scalloping on the right side (Fig. 1).

On the basis of the clinical and radiographic characteristics, diagnosis of bilateral nasolabial cyst was established.

**Figure 2:**
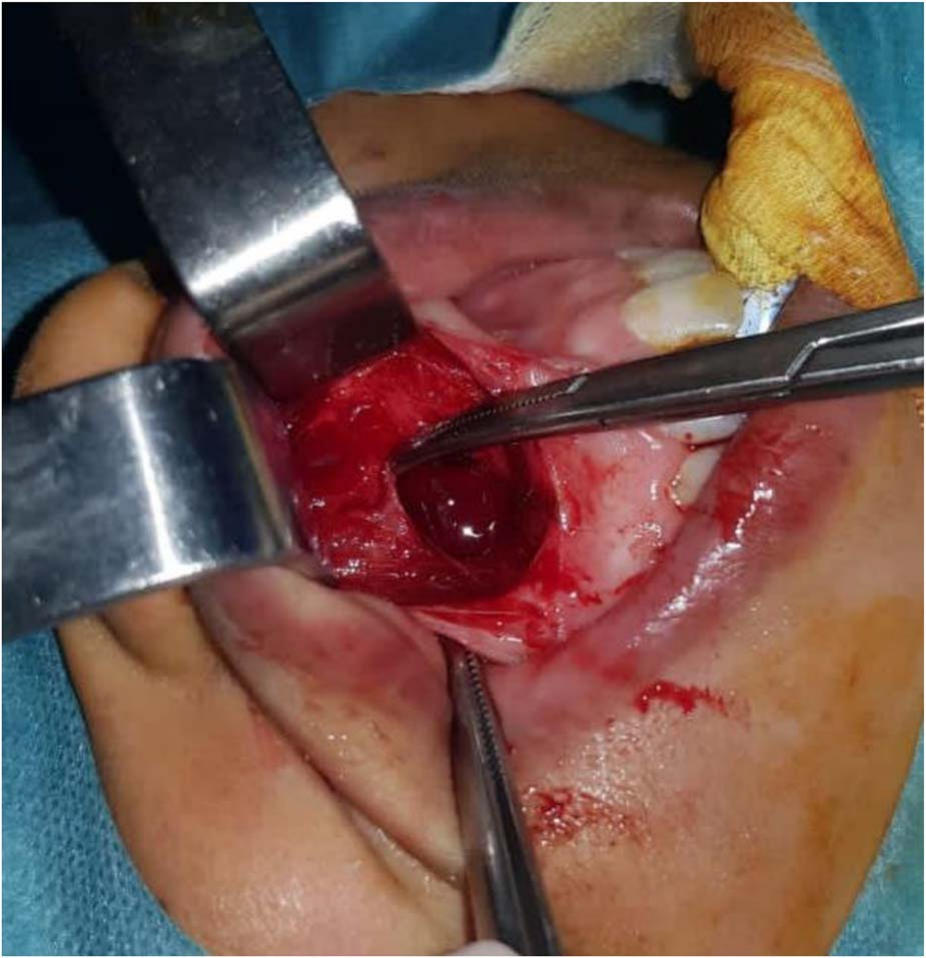
Intraoperative appearance of thecyst.

The patient was subjected to enucleation of the bilateral cysts under general anesthesia (Fig. 2). A full-thickness upper vestibular incision was performed in the canine-to-canine region. The incision was placed in the upper vestibular sulcus 1 cm above the upper margin of the attached gingival. The periosteum was then elevated using a periosteal elevator. Two smooth, round cystic swellings bellow the nasal floor were exposed. The cysts were bluntly dissected. The surgical wound was closed using 4/0 polyglactin interrupted sutures.

Histopathological examination of the specimens showed respiratory (ciliated pseudostratified columnar epithelium) with goblet cells compatible with nasolabial cysts.

**Figure 3:**
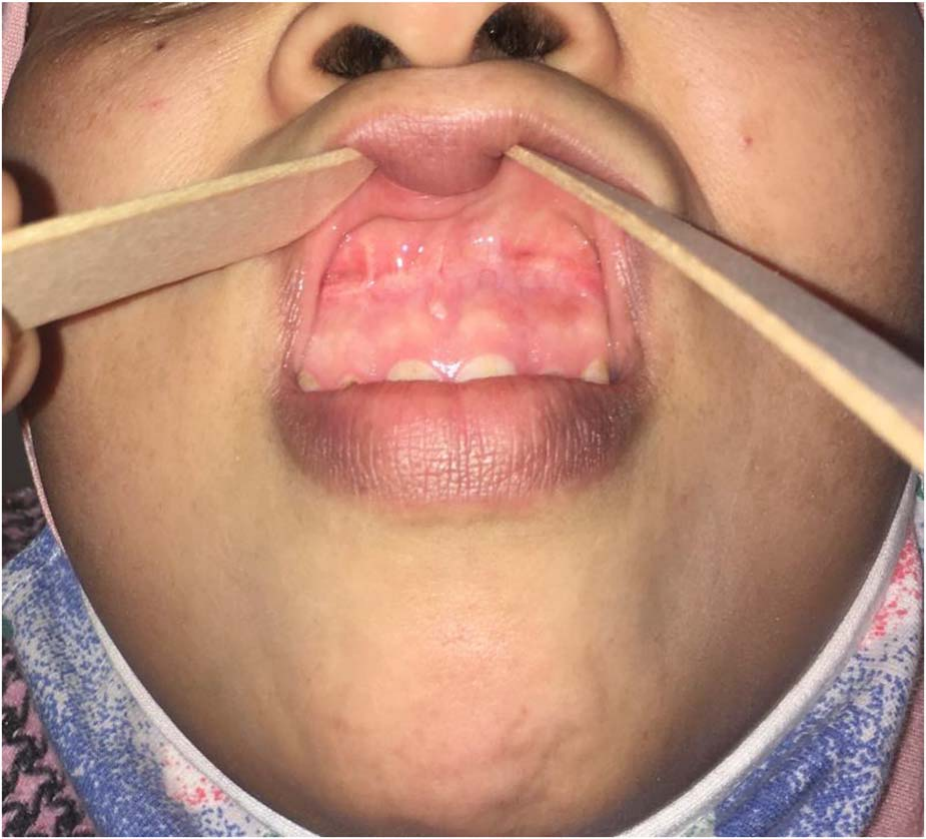
Appearance 8 months after surgical treatment.

No sign of recurrence was observed 8 months after surgery (Fig. 3).

## DISCUSSION

Maxillary cysts are divided into two major groups: odontogenic cysts and non-odontogenic cysts. The first group is characterized by specific odontogenic markers, histological similarities with odontogenic structures and anatomical considerations [3].

Nasolabial cyst is a soft tissue cyst that is often located submucosally in the anterior nasal floor [2]. These cysts account for 0.7% of all non-odontogenic cysts [4].

The etiology of nasolabial cysts is still debated. The cyst was initially thought to originate from the retention cysts arising from inflamed mucous glands. The current prevailing theory is that the cysts are the remnants of embryonic nasolacrimal duct tissue [1].

This hypothesis is reinforced by the fact that nasolacrimal ducts are lined with pseudostratified columnar epithelium, the same epithelium frequently found in nasolabial cysts [1].

Most cases occur between the fourth and fifth decades of life. Male-to-female ratio is 1:3.6 [5]. Nasolabial cyst commonly arises on the left side. Bilaterality is reported in only 10% of cases in the literature [4].

The diagnosis of nasolabial cyst is essentially clinical. The difficulty in diagnosing this lesion is their rarity. It may be asymptomatic, however, it usually manifests as swelling in the gingivolabial sulcus leading to facial asymmetry. Pain is an uncommon sign denoting an infected cyst. Our patient experienced chronic nasal obstruction. Few cases of nasolabial cysts presenting in this manner have been reported in the literature [6]. Bimanual palpation of the nasal floor and labial sulcus typically reveals a soft, fluctuant mass [7].

The differential diagnosis is made with odontogenic lesions such as follicular, periodontal and residual cysts, canine space abscess and salivary gland neoplasms. Dermoid and epidermoid cysts should also be considered in the differential [5].

Radiographic films, computed tomography (CT) and magnetic resonance imaging (MRI) have been used to aid in the diagnosis of nasolabial cysts [1]. Radiographs cannot diagnose this soft lesion unless there is significant maxillary bone erosion [8]. CT scan is the imaging modality of choice for the diagnosis of nasolabial cysts. In a literature review involving 311 patients conducted by Sheikh *et al*., 170 (54.7%) patients were diagnosed with CT scan. It typically reveals a homogeneous, cystic lesion anterior to the piriform aperture [5]. Scalloped bone resorption is common in large lesions as was the case in our patient [1]. MRI, although efficient, is less used in clinical practice.

On histopathological examination, The cyst consists of respiratory epithelium, stratified ciliated cylindrical epithelium with goblet cells or pseudostratified ciliated cylindrical [1]. Squamous metaplasia may occur in infected cysts [9].

Treatment for nasolabial cysts is generally the removal of cyst trough an intra-oral sublabial excision under local or general anesthesia [1]. Other techniques have been reported in the literature, including cauterization, injection of sclerosing agents, aspiration and incision and drainage. However, these methods are associated with high recurrence rates [1, 5].

An endoscopic transnasal approach have been recently described [10]. In this technique, an incision is made over the cyst in the nasal floor and the cyst is subsequently marsupialized. This approach has the advantage of a shorter operative time as well as a reduced overall rate of complications [1].

Otolaryngologist should consider the diagnosis of nasolabial cysts in the presence of chronic nasal obstruction. This will prevent delay in diagnosis and ensure proper treatment of the disease.

## CONFLICT OF INTEREST STATEMENT

None declared.

## FUNDING

The authors declared that this study has received no financial support.

## ETHICAL APPROVAL

Written informed consent for publication of their clinical details and/or clinical images was obtained from the patient’s parent. Ethical approval has been exempted by our institution.
